# Metabolite Identification of a Novel Anti-Leishmanial Agent OJT007 in Rat Liver Microsomes Using LC-MS/MS

**DOI:** 10.3390/molecules27092854

**Published:** 2022-04-30

**Authors:** Maria Eugenia Rincon Nigro, Ting Du, Song Gao, Manvir Kaur, Huan Xie, Omonike Arike Olaleye, Dong Liang

**Affiliations:** Department of Pharmaceutical Science, Texas Southern University, Houston, TX 77004, USA; marie.rincon.nigro@gmail.com (M.E.R.N.); du.ting@tsu.edu (T.D.); song.gao@tsu.edu (S.G.); m.kaur3826@student.tsu.edu (M.K.); huan.xie@tsu.edu (H.X.); omonike.olaleye@tsu.edu (O.A.O.)

**Keywords:** LC-MS/MS, liver microsomes, metabolic stability, UGT, CYP450

## Abstract

The purpose of this study was to identify potential metabolic pathways and metabolites of OJT007, a methionine aminopeptidase 1 (MetAP1) inhibitor. OJT007 is a novel drug with potent antiproliferative effects against Leishmania Major. We conducted in vitro Phase I oxidation and Phase II glucuronidation assays on OJT007 using rat liver microsomes. Four unknown metabolites were initially identified using a UPLC-UV system from microsomal incubated samples. LC-MS/MS analysis was then used to identify the structural characteristics of these metabolites via precursor ion scan, neutral loss scan, and product ion scan. A glucuronide metabolite was further confirmed by β-glucuronidase hydrolysis. The kinetic parameters of OJT007 glucuronidation demonstrated that OJT007 undergoes rapid metabolism. These results demonstrate the liver’s microsomal ability to mediate three mono-oxidated metabolites and one mono-glucuronide metabolite. This suggests hepatic glucuronidation metabolism of OJT007 may be the cause of its poor oral bioavailability.

## 1. Introduction

Leishmaniasis is a neglected disease caused by protozoan parasites carried through the bite of phlebotomine sand flies. It is an endemic disease with an estimated 70,000 to 1 million cases per year and more than one billion at risk of infection [1]. Clinical manifestations of leishmaniasis range from self-healing cutaneous lesions with probable mucosal spread to severe visceral forms that can be lethal if untreated [2]. Currently, there is no human vaccination available to combat *Leishmania* infections, and treatment is primarily dependent on chemotherapy [3].

Despite recent advances in new drugs discovery, the therapeutic management of this ancient disease remains a challenge especially with increasing levels of drug resistance. The current treatment of Leishmaniasis is largely based on repurposed drugs [4]. This has led to numerous drawbacks, due to high toxicity, limited availability, long-term treatment protocols, high cost, and poor patient compliance. Moreover, the emergence of multi-drug-resistant strains has emphasized the imperative need to develop novel anti-leishmaniasis agents [5]. To overcome the challenges posed by current treatment therapy, it is crucial to discover safer and more effective chemical scaffolds to target novel mechanisms of action.

Methionine aminopeptidase (MetAP), a di-nuclear metalloprotein, is required for bacterial growth and survival [6,7]. It is responsible for the excision of the initiating N-terminal methionine from nascent proteins during translation [8,9]. The removal of the N-terminal methionine is crucial for maturation, biological activation, subcellular localization, and the degradation of proteins [10,11]. All organisms, from bacteria to eukaryotes, have MetAP. There are two major classes of MetAP: MetAP1 and MetAP2. Eukaryotes express both types, while prokaryotes have homologs of either MetAP1 (eubacteria) or MetAP2 (archaebacteria) [8,12,13]. Genetic studies have revealed the physiological importance of MetAP enzyme activity, as the deletion of the MetAP1 gene causes lethality in prokaryotes [11,12]. The essential role of MetAP makes this enzyme a promising target for the discovery and development of novel antileishmanial agents. 

Previously, using high throughput and chemical genomic approaches, OJT007 was discovered as a potent inhibitor of *Mycobacterium tuberculosis* MetAP1c [7]. More recently, OJT007 was identified and characterized as a novel inhibitor of *L. major* amastigotes and promastigotes with an effective concentration (EC_50_) of 0.5 μM and no cytotoxicity against host cells. Furthermore, the drug has demonstrated oral efficacy in a preclinical mouse model study, with no resulting cardiac or hepatic damage [13]. These results further indicate OJT007 as a promising oral chemotherapeutic agent for the treatment of cutaneous leishmaniasis.

Even though OJT007 has excellent potency, its successful clinical application may be limited by the low oral bioavailability shown in rat models, 10.9%. In addition, our animal studies in rats have found that OJT007 is extensively metabolized with less than 1% parent drug recovered in urine (Unpublished Data). To better understand the in vivo metabolism and disposition of OJT007, in the present study, we aimed to explore the role of liver microsomal CYP450 and UGT enzymes to elucidate possible mechanisms causing bioavailability attenuation. 

The identification and quantification of metabolites are essential during drug development phases to understand the behavior of drugs in humans. An early understanding of the metabolite profile may increase drug safety, prevent drug–drug interactions, and discover the role of metabolites in the overall pharmacology of the drug. This information may aid in lowering attrition rates and overall development costs [14,15]. The Food and Drug Administration (FDA) recommends metabolite profiling for metabolites that account for more than 10% of the total drug-related components [16].

In this study, we used an LC-MS-based approach to investigate the metabolic composition of OJT007. MS offers quantitative analyses of metabolites with higher sensitivity and resolution [17]. In MS-based metabolomic research, the most often used analytic tools are gas chromatography mass spectrometry (GC–MS) and liquid chromatography mass spectrometry (LC–MS). LC–MS has an advantage over GC–MS because metabolites separated by LC or ion chromatography do not need chemical derivatization [18]. As a result, LC-MS can work with a variety of column chemistries and a greater spectrum of compounds. With the development of ultra-performance liquid chromatography (UPLC) and extremely accurate mass spectrometry (MS), the use of UPLC–MS in metabolomic investigations has expanded substantially [18,19]. LC-MS/MS Q1 multiple ion (Q1 MI), neutral loss (NL), and product ion (MS2) scans were used in this study to confirm the identity of CYP450 and glucuronide metabolites. In addition, we evaluated CYP450-mediated metabolic stability and the rate of formation of OJT007 glucuronide.

## 2. Results and Discussion

### 2.1. Full Mass Scan and Product Ion Scan Spectra for Matrix Free OJT007

**Figure 1:**
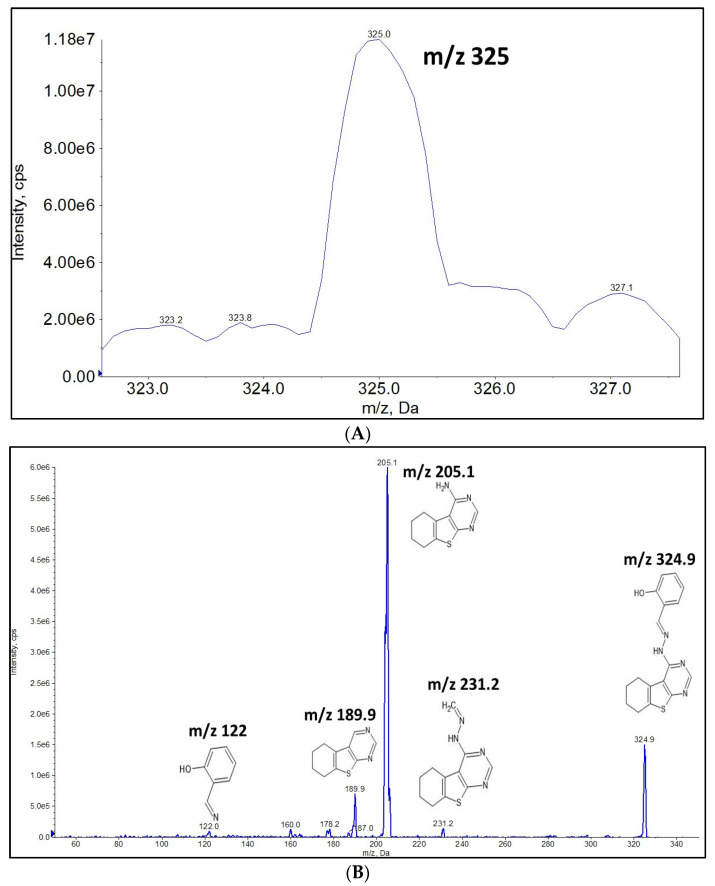
(**A**) Q1 MS Spectra for OJT007. (**B**) MS2 Spectra for OJT007.

The monoisotopic mass for OJT007 is 324.2 Da. Due to the presence of amine moieties, OJT007 showed more response in positive mode (*m*/*z* 325) as a protonated molecule [M+H]^+^. In Q1 MS scan, the presence of OJT007 was confirmed by the protonated molecular ion at *m*/*z* 325 [M+H]^+^ (Figure 1A). An MS2 scan of matrix free OJT007 (Figure 1B) identified a protonated molecular ion at *m*/*z* 325 [M+H]^+^. The most intense fragment ion corresponded to *m*/*z* 205. The second most abundant fragment corresponded to *m*/*z* 189.9. The fragment at *m*/*z* 122 corresponds to the protonated methanimidoyl phenol moiety in OJT007.

### 2.2. Metabolism of OJT007 in CYP and UGT Reaction Systems

CYP450 is a superfamily of enzymes that are responsible for metabolism of the majority of marketed drugs. UDP-glucuronosyltransferases (UGTs) are a family of enzymes that catalyze glucuronidation reaction. CYPs and UGTs represent more than 80% of the metabolic pathways of drugs [20]; thus, we evaluated the CYP and UGT-mediated metabolisms of OJT007.

#### 2.2.1. CYP Mediated Phase I Metabolism 

**Figure 2:**
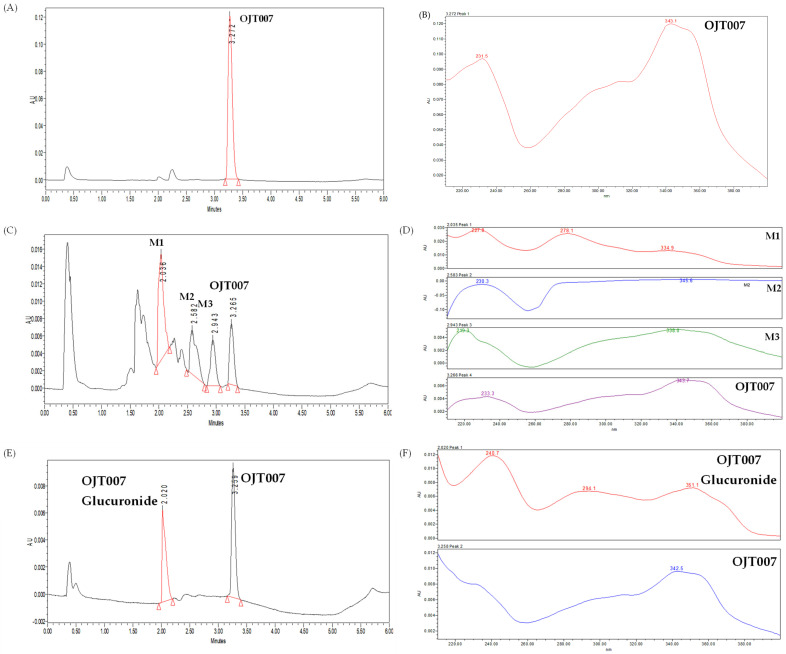
(**A**) Representative chromatogram and (**B**) UV Spectra of OJT007 before microsomal incubation OJT007. (**C**) Representative chromatogram and (**D**) UV spectra of OJT007 and oxidation metabolites after microsomal incubation. (**E**) Representative chromatogram of OJT007 and glucuronidation metabolite. (**F**) UV spectra of OJT007 and glucuronide metabolite.

OJT007 has a retention time of 3.27 min in UPLC-UV analysis (Figure 2A). After incubation with hepatic rat microsomes, three additional peaks were observed at retention times of 2.036, 2.583, and 2.943 min in UPLC analysis (Figure 2C). The UV spectra of these additional peaks displayed similar patterns to that of OJT007 (Figure 2D). 

##### Phase I Metabolites Identification by LC-MS/MS 

**Figure 3:**
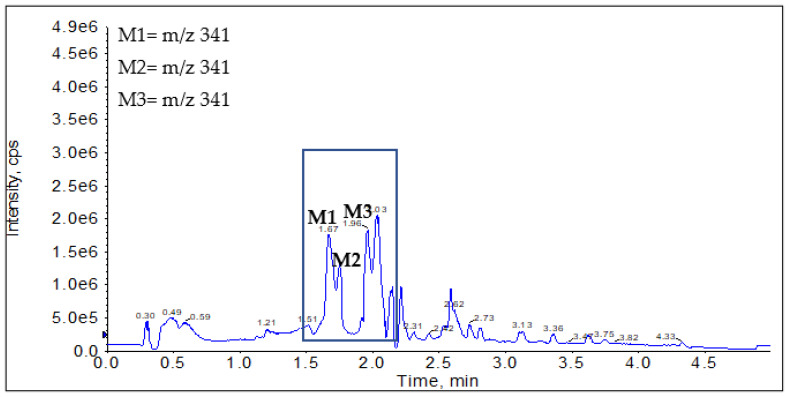
Q1MI chromatogram for *m*/*z* 341 obtained from NADPH activated liver microsomes. M1 retention time: 1.67 min; M2 retention time: 1.76 min; M3 retention time: 1.96 min.

LC-MS/MS analysis was used to elucidate the structures of the newly eluted peaks. Targeted metabolite identification takes advantage of the extensive understanding of metabolic enzymes and the pathways to which they could contribute. Hydroxylation reactions are very common in drugs containing aromatic rings or aliphatic functional group (saturated or unsaturated) in their chemical structure [21]. Based on the protonated molecular ion for OJT007 *m*/*z* 325 [M+H]^+^, we conducted a Q1 MI scan for *m*/*z* 341 [M+16+H]^+^. Q1MI (Q1 Multiple Ion) scan (Figure 3) identified a protonated molecular ion at *m*/*z* 341 [M+16+H]^+^, indicating hydroxylation at 1.67, 1.76, and 1.96 min.

**Figure 4:**
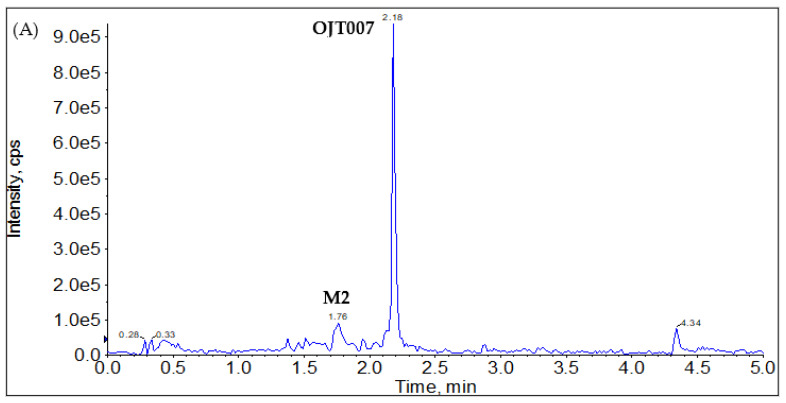

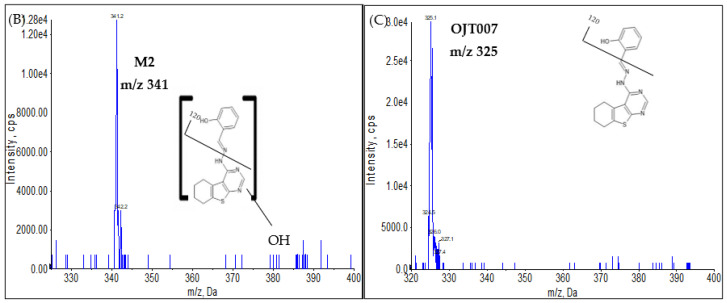
(**A**) Neutral loss (NL) of 120 Da Chromatogram (**B**) Spectra for M2 (retention time: 1.76 min) (M2). (**C**) Spectra for OJT007 (retention time: 2.18 min).

In neutral loss scans, ions are scanned on Q1, fragmented in Q2, and scanned for changes in mass which correspond to a set neutral loss in Q3. In other words, neutral loss scan displays the spectrum of all parent ions that lose a selected neutral loss fragment. From the product ion spectrum of OJT007 standard (Figure 1), the main product ion is *m*/*z* 205, corresponding to the loss of 120 Da. Thus, we performed a neutral loss scan for 120. This procedure (Figure 4A) indicated the presence of OJT007 itself (retention time: 2.18 min) with an *m*/*z* of 325 (Figure 4B) in addition to a metabolite, which eluted at 1.76 min with an *m*/*z* of 341 (Figure 4C). This finding confirmed that the peak eluting at 1.76 min corresponds to a mono-oxidative metabolite. In addition, this suggests that the oxidation occurs in the 5,6,7,8-Tetrahydro [1] benzothieno [2,3-d]pyrimidin-4-amine portion of the molecule.

**Figure 5:**
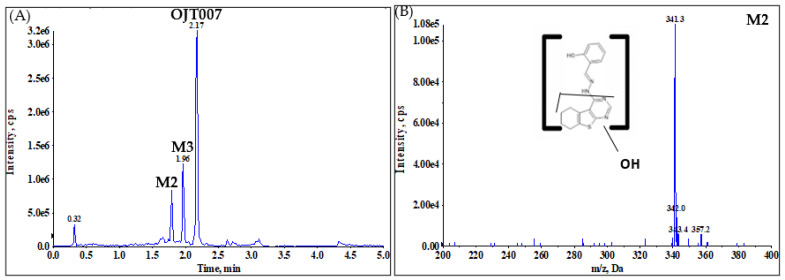

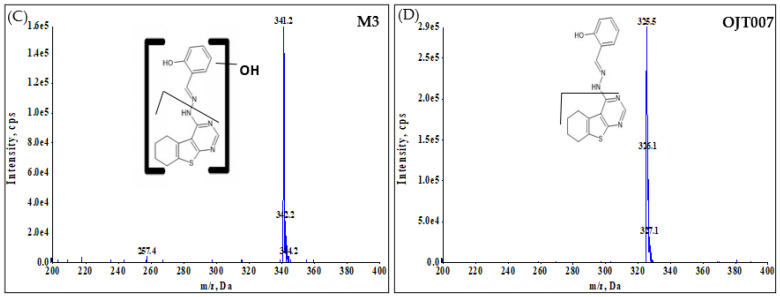
(**A**) Neutral loss of 136 Da chromatogram. (**B**) Spectra peak at 1.76 min (M2). (**C**) Spectra for peak at 1.96 min (M3). (**D**) Spectra for peak at 2.17 min (OJT007).

A NL scan (Figure 5) was then performed for 136 Da (120 plus 16) to screen for metabolites obtained by hydroxylation on the neutral fragment. Neutral loss scan evidenced three peaks eluting at 1.76, 1.96, and 2.17 min. NL spectra for the peaks eluting at 1.76 (Figure 5B) and 1.96 (Figure 5C) showed ions with *m*/*z* 341, which is 16 Da higher than the addition ion of OJT007, confirming both peaks as metabolites. Furthermore, the neutral loss scan suggests that oxidation for the metabolite eluting at 1.96 occurs in the phenol portion of the molecule. It is possible that the same neutral loss that produced the peak at 2.17 min (parent molecule), gave rise to the peak eluting at 1.76 min. 

**Figure 6:**
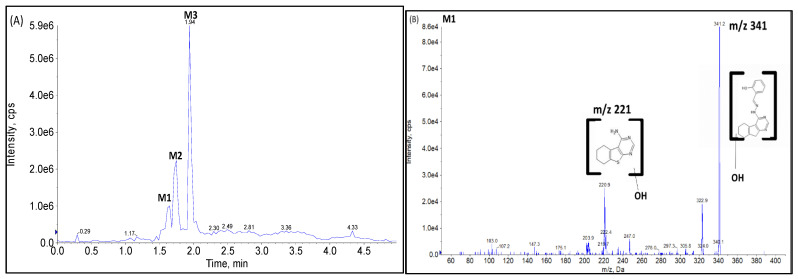

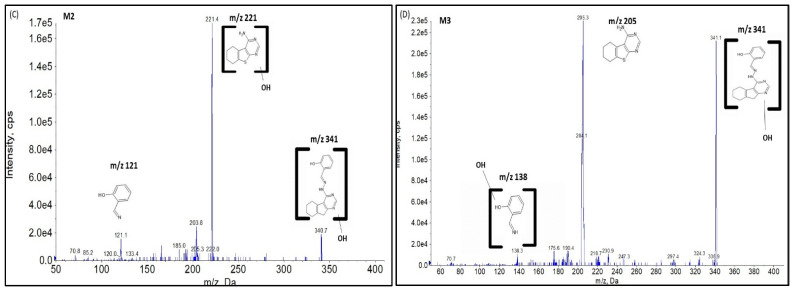
(**A**) Product ion scan Chromatogram *m*/*z* 341. (**B**) MS2 Spectra of M1 (retention time: 1.66 min). (**C**) MS2 Spectra of M2 (retention time: 1.76 min). (**D**) MS2 Spectra of M3 (retention time: 1.96 min).

**Figure 7:**
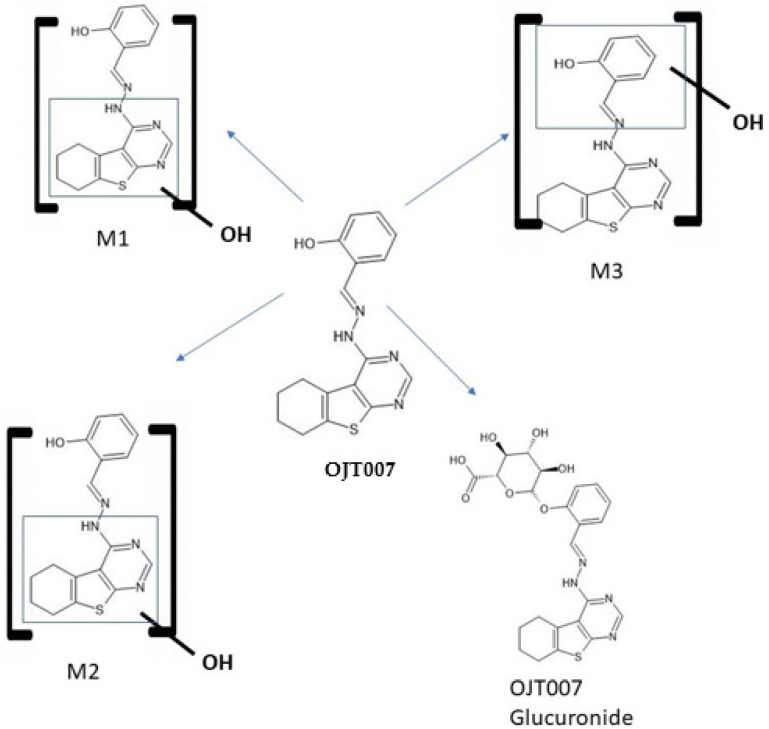
Proposed Metabolic pathway of OJT007 in rat liver microsomes. M1, M2, and M3 represent mono-oxidation metabolite (*m*/*z* 341). OJT007 glucuronide represent glucuronidation metabolite (*m*/*z* 501).

As a final confirmation step, product ion analysis (MS2) was performed. The fragmentation pattern of the metabolites was compared to the parent compound. An MS2 scan of microsomal samples (Figure 6A–D) showed a protonated molecular ion for the metabolites at *m*/*z* 341 [M+16+H]^+^, which is 16 Da higher than that of OJT007 (*m*/*z* 325 [M+H]^+^), indicating hydroxylation. The spectra for the peak eluting at 1.66 (Figure 6B) shows a major fragment at *m*/*z* 221, which is 16 Da higher than the major fragment for OJT007. The MS2 spectrum for the peak eluting at 1.76 min (M2) showed major fragment ions at *m*/*z* 221 and 121. The presence of the product ion at *m*/*z* 121 suggests that methanimidoyl phenol moiety is unaltered. The fragment ion at *m*/*z* 221 had a mass shift of 16 Da from the corresponding product ion of OJT007, suggesting that hydroxylation occurred on the benzothieno pyrimidine moiety of the molecule (Figure 6C). The MS2 spectra for the peak eluting at 1.94 (Figure 6D) show a major fragment at *m*/*z* 205, similarly to the major fragment ion of OJT007. Likewise, the spectra for the peak eluting at 1.94 min show a fragment at *m*/*z* 138, which is 16 Da higher than the corresponding product ion of OJT007 at *m*/*z* 122 fragment, suggesting that hydroxylation potentially occurred in the phenol ring. Based on these spectra, we have proposed possible metabolite structures for M1, M2, and M3 in Figure 7.

##### Metabolic Stability for Oxidation Metabolites

In a typical microsomal stability assay, protein concentrations are kept below 2 mg protein/mL (to prevent nonspecific binding) with incubation times below 1 h (to prevent loss of enzyme activity). In addition, measuring the depletion of the parent drug entails at least 10–15% turnover to make a reliable measurement. This requirement is based on the bioanalytical method having accuracy and precision values within that range [22].

In our study, OJT007 displayed no significant turnover of the parent drug with enzyme concentrations below 10 mg protein/mL; thus, we used a high enzyme concentration. Furthermore, substrate depletion was observed in the incubation without cofactor, suggesting chemical instability at buffer pH. Likewise, it could indicate possible non-NADPH-dependent enzymatic degradation. For screening purposes, the metabolic half-life was determined using a single time point analysis based on first-order reaction kinetics [23]. The equation for the calculation of half-life from the % remaining from the parent at a given time is shown in Equation (3). The half-life for OJT007 is 1.28 h at 10 mg of protein/mL enzyme concentration, suggesting phase I metabolism is relatively slow [24]. The CYP metabolic screening results suggested that Phase I metabolism, although happening, may not be playing an important role in OJT007 metabolism.

#### 2.2.2. UGT Mediated Glucuronidation Metabolism

Rapid conjugation in the intestine and liver is primarily responsible for the poor oral bioavailability of phenolics. Glucuronidation of phenolics generally occurs at the nucleophilic –OH attached to the aromatic ring (O-glucuronidation). In an OJT007 molecule, there is one hydroxyl group that may serve as the glucuronidation site. Thus, we evaluated glucuronidation in OJT007.

After microsomal incubation, an additional peak was observed at 2.020 min (Figure 2E). The UV spectra of this newly eluted peak were similar to that of OJT007 (Figure 2F), suggesting that the skeleton of the additional peak was similar to that of OJT007. This newly eluted peak is tentatively identified as OJT007 glucuronide.

##### Glucuronide Identification by LC-MS/MS 

**Figure 8:**
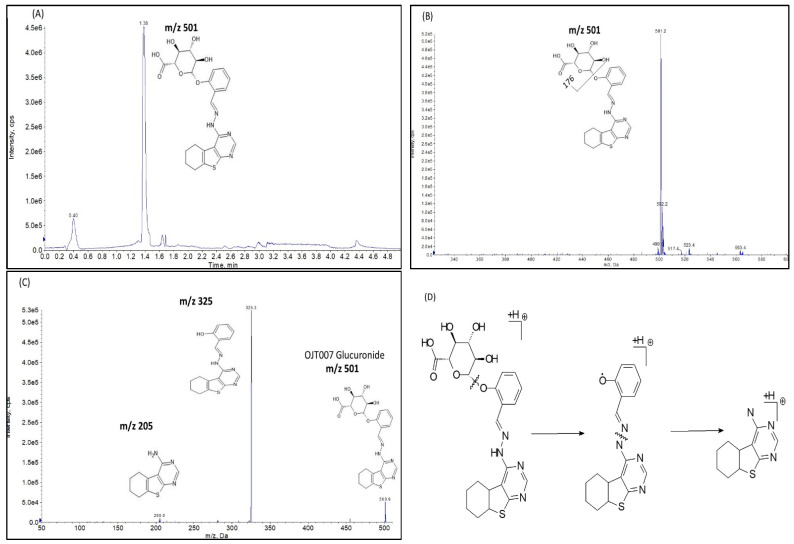
(**A**) Representative Q1MI chromatogram, OJT007 Glucuronide retention time 1.38 min. (**B**) Neutral loss scan of 176 Da spectra. (**C**) Product ion scan spectra *m*/*z* 501. (**D**) Fragmentation pattern for OJT007 glucuronide.

Q1 multiple ions (Q1MI), product ion (MS2) scan, and Neutral loss (NL) scan in positive mode were used to identify the metabolite. In the Q1MI scan, Q1 works in selected ion monitoring (SIM) by selecting a specific *m*/*z* value. The Q1MI scan evidenced a new peak eluting at 1.38 min (Figure 8A). The newly eluted peak had a protonated molecular ion at *m*/*z* 501.2 [M+176+H]^+^, which is 176 Da higher than that of OJT007 at *m*/*z* 325. Figure 8B shows the spectra obtained after a neutral loss 176. After NL of 176 Da, a new peak eluted at 1.38 min, which had a precursor ion at *m*/*z* of 501, as shown in the spectra, which confirms the presence of a glucuronide.

The MS2 spectra (Figure 8C) show a protonated molecular ion of the conjugation metabolite at *m*/*z* 501.2. This is 176 Da higher than that of OJT007 at *m*/*z* 325, indicating the presence of glucuronic acid. Moreover, the spectra showed major fragment ions at *m*/*z* 325.1 and 205. The fragment *m*/*z* 325.1 is similar to the mass of the addition ion of OJT007 at *m*/*z* 325. The ion observed at *m*/*z* 325.1 was caused by loss of the glucuronic acid moiety (176 Da) from the protonated molecular ion at *m*/*z* 501. On the other hand, the fragment ion at *m*/*z* 205 is similar to the major fragment ion for OJT007. These spectra data confirm that the newly eluted metabolite corresponds to a mono-glucuronidated OJT007. 

In this paper, we have demonstrated a strategy for oxidation and glucuronidation metabolite identification using a triple quadrupole mass spectrometer. The strategy was based on screening the metabolites via Q1 MI, neutral loss, and product ion scan based on the results from parent drug product ion scan. We demonstrated the feasibility of the strategy using rat liver microsomes, and we identified three Phase I and one Phase II metabolites of OJT007. This approach may be useful in studying metabolic process of other new drug candidates that are primarily metabolized via hepatic microsomal enzymes.

##### Glucuronide Hydrolysis by β-Glucuronidase 

**Figure 9:**
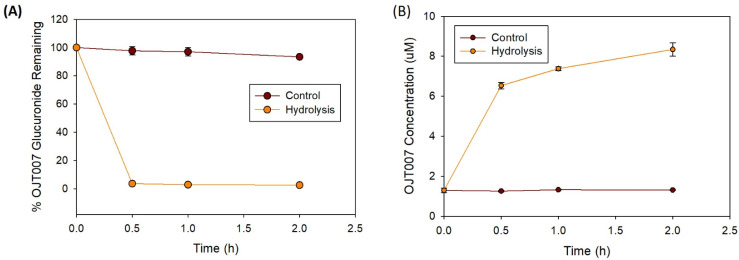
Hydrolysis of OJT007 glucuronide generated by rat liver microsomes with *E.Coli* β-glucuronidase. (**A**) Remaining glucuronide metabolite after hydrolysis. (**B**) OJT007 concentration after hydrolysis. Each data point indicated the mean ± SD.

Figure 9 shows the hydrolysis of the OJT007 glucuronides, which were produced from liver microsomes by β-glucuronidases. After incubating with β glucuronidases from *E. coli* for two hours, OJT007 concentrations increased at least 5-fold when compared with the control (Figure 9A), whereas OJT007 glucuronide residuals dramatically decreased (Figure 9B). These chemical data confirm that the newly eluted peak is OJT007 glucuronide.

##### Conversion Factors (K) of Extinction Coefficient

New chemical entities seldom have commercially available authentic standards for their metabolites. This situation becomes an important hurdle in enzyme kinetic studies because, ideally, all quantification must be conducted against a reference standard. Overcoming this difficulty using LC-MS/MS is not advisable. The addition of polar ionizable groups such as glucuronides during metabolism dramatically impacts the molecule’s ionization resulting in an over- or underestimation of metabolism [25]. Quantification of metabolite by LC-UV is a more sensible approach because each compound has an extinction coefficient. Furthermore, extinction coefficients are a function of its chemical moieties or chromophores. If these structural motifs do not change significantly in the metabolite compared to the parent molecule, it is reasonable to assume that the metabolite’s extinction coefficient has not changed. In this case, metabolite concentrations can be compared to the parent molecule using the standard curve built for the parent molecule. Many studies assume that molar extinction coefficients remain the same as their corresponding parent; therefore, quantification was accomplished in each of those cases using the calibration curve of the parent drug [26]. More precise estimations can be accomplished by determining conversion factor (K), which is the ratio between the molar extinction coefficient of the metabolite and the parent drug [27,28]. To accomplish this task, the glucuronidation reaction was performed at three different concentrations (2, 10, and 50 μM) to calculate average K values. The results showed that the K value for OJT007 glucuronide was 1.06. This conversion factor was used to calculate the concentration of OJT007 glucuronide using the standard curve for OJT007.

##### Kinetics of OJT007 Glucuronidation

**Figure 10:**
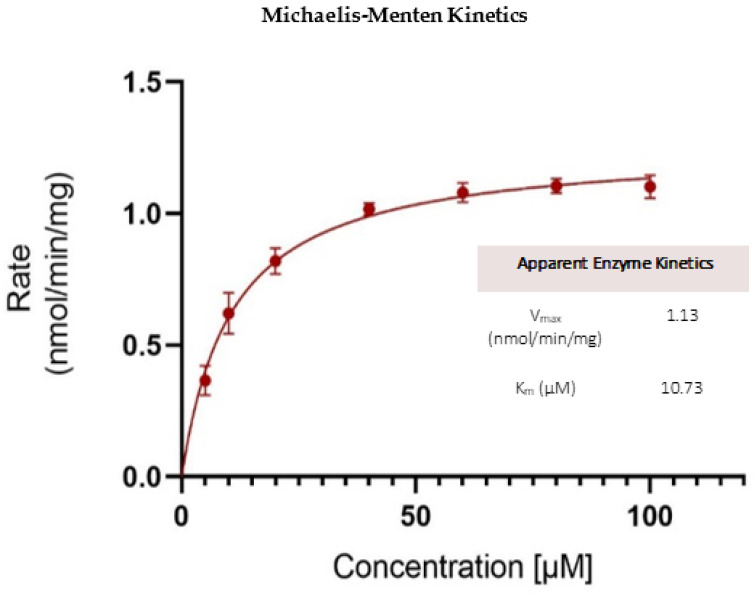
Apparent enzyme kinetics for OJT007 glucuronide.

The OJT007 glucuronidation rate was determined by measuring the amount of metabolite formed in microsomes divided by the reaction time and protein concentrations. The substrate concentration evaluated ranged from 1.25 to 100 μM, with a protein concentration of 0.035 mg of protein/mL and an incubation time of 60 min. The most appropriate model was selected based on the visual inspection of the Eadie-Hofstee plot. Quantitative analysis showed that the substrate concentration–glucuronidation velocity curves followed Michaelis–Menten kinetics (Figure 10). The maximal metabolic rate (V_max_) and the Michaelis Menten constant (K_m_) were 1.125 nmol/min/mg and 10.73 μM, respectively. 

The rate of OJT007 glucuronidation was rapid via liver microsomes with an intrinsic clearance value similar to those of isoflavones, a class of compounds with limited oral bioavailability due to extensive glucuronidation metabolism [9]. It is possible that extensive glucuronidation may have implications in the in vivo oral bioavailability of OJT007.

## 3. Materials and Methods

### 3.1. Materials

OJT007 (purity > 90%) was purchased from MolPort (Riga, Latvia). Formic acid and dimethyl sulfoxide (DMSO) were procured from Sigma-Aldrich (St. Louis, MO, USA). LC-MS grade water and acetonitrile were purchased from J.T. Baker Chemical Co. (Phillipsburg, NJ, USA). LC-MS/UPLC grade Methanol was acquired from Mallinckrodt Baker (Phillipsburg, NJ, USA). The blank rat plasma was purchased from Innovative Research (Novi, MI, USA). The rat (Sprague-Dawley) pooled liver microsomes, male, were purchased from Corning Gentest (Corning, New York, NY, USA). The NADPH regenerating system was purchased from Corning Gentest (Corning, New York, NY, USA).

### 3.2. Sample Preparation

Test samples were obtained by incubating OJT007 in rat liver microsomes to generate metabolites. Samples measuring 100 μL were treated with 50 μL acetonitrile containing 0.6% formic acid. The samples were vortexed for 30 s, centrifuged at 14,000 rpm for 15 min, and the supernatant was injected for analysis. A stock solution of OJT007 was prepared by dissolving the solid compound in 5% DMSO and 95% methanol at a concentration of 1 mg/mL. The standard curve samples (100 μL) containing OJT007 were prepared in a solution or phosphate buffer and quenched with 50 μL acetonitrile containing 0.6% formic acid. The standard curve points were centrifuged at 14,000× *g* rpm for 15 min, and the supernatant was injected into UPLC for quantification.

### 3.3. LC-MS/MS Method

#### 3.3.1. Chromatography

UPLC analysis was accomplished using a Shimadzu Nexera X2 UHPLC system (Columbia, MD, USA). Previously reported chromatographic conditions were adopted in this study [29]. Briefly, OJT007 is a lipophilic compound; as such, chromatographic separation was achieved using an Acquity UPLC BEH C18 column (50 × 2.1 mm, 1.7 μm) with a gradient mobile phase at flow rate of 0.4 mL/min. The sample injection volume was 10 μL, and the mobile phase consisted of 0.1% *v/v* formic acid in water (A) and 0.1% *v/v* formic acid in acetonitrile (B). Gradient elution was employed with 5% to 98% B from time 0 to 1.8 min, and it was kept constant at 98% B for 1.7 min; then, 98% B was changed to 5% B from 3.5 to 3.7 min and kept constant at 5% from 3.7 min to 5 min.

#### 3.3.2. Mass Spectrometry

MS/MS analysis was performed on a 4000 QTRAP^®^ triple quadrupole mass spectrometer system equipped with a Turbo Ion Spray ion source (AB Sciex. Redwood City, CA, USA). The mass spectrometer was operated in positive ion electrospray (ESI) mode. The general mass spectrometric source and compound dependent parameter conditions were set as follows based on a previously developed methods with modifications [29]: Briefly, the compound dependent parameters were optimized based on the parent compound’s (OJT007) ionization efficiency and fragmentation pattern. By using the Q1MI scan mode, the XIC signal of the protonated molecular ion at *m*/*z* 325 was monitored, ramping the DP values from 0 to 400 V. The best signal intensity was found with DP of 105 V, because it effectively eliminates ion clusters. Likewise, EP was set at 10 V. In a subsequent experiment, using product ion scan, the fragmentation of the precursor ion was evaluated by ramping CE values from 10 to 100 V. The optimal CE value for the formation of the most intense fragment (*m*/*z* 205) was 30 V; however, in this experiment, we increased CE to 35 V to obtain more fragmentation of the most abundant ion (*m*/*z* 205) for aiding in structural elucidation. Similarly, the collision exit potential (CXP) was optimized to 11 V by ramping the value from 5 to 100 V to ensure optimal sensitivity. The source parameters were optimized in order to obtain the most suitable conditions for the analyte while ensuring signal stability and sensitivity. Analyst^®^ Software 1.6.2 (Redwood City, CA, USA) was used to control the LC-MS/MS system and to analyze the data.

### 3.4. UPLC Method

To prevent ion source contamination due to the buffered in vitro metabolism experiments and to quantitate metabolites, a UPLC analytical method was developed. The UPLC method was based on previously reported LC conditions with modifications in gradient time to ensure the appropriate separation of parent and metabolites [29]. Briefly, the UPLC system consisted of Waters Acquity UPLC with photodiode array detector and Empower software, column, BEH C18, (1.7 μm, 2.1 × 50 mm. The mobile phase consisted of 100% acetonitrile (B) and 0.1% formic acid in water (A). Gradient elution was employed and optimized to ensure appropriate chromatographic retention and separation of parent compounds and metabolites. The gradient elution consisted of 5% to 98% B from time 0 to 2.8 min and was kept constant at 98% B for 1.2 min, then 98% B was changed to 5% B from 4 to 5.2 min and kept constant at 5% from 5.2 min to 6 min. UV detection wavelength was 343 nm; the injection volume was 10 μL. 

### 3.5. In Vitro Metabolic Studies—CYP Reaction System

An NADPH regenerating system consists of solution A and solution B, which after mixing generate the NADPH needed for oxidation reactions. Solution A contains 26 mM NADP+, 66 mM glucose-6-phosphate, and 66 mM magnesium chloride in water, while solution B containing 40 U/mL glucose-6-phosphate dehydrogenase in 5 mM sodium citrate was used for generating NADPH. The in vitro cytochrome P450 phase I oxidation metabolism of OJT007 was evaluated according to previously described incubation procedures [30]. Briefly, rat liver microsomes (final concentration 10 mg of protein/mL), 50 mM phosphate buffer at pH 7.4 (KPI), test compound (50 μM), 30 μL NADPH regenerating solution A, and 6 μL NADPH regenerating solution B were incubated for 28 h at 37 °C (600 μL total assay volume). Aliquots of 100 μL were taken at different time points and quenched with 50 μL of ice-cold acetonitrile containing 0.6% formic acid. The samples were vortexed for 30 s, centrifuged at 14,000× *g* rpm for 15 min, and the supernatant was injected into UPLC system for analysis. A negative control was prepared the without addition of NADPH.

### 3.6. In Vitro Metabolic Studies—UGT Reaction System

The in vitro uridine glucuronosyltransferase (UGT) phase II glucuronidation of OJT007 was evaluated according to previously described incubation [31]. Rat liver microsomes (final concentration of 0.035 mg protein/mL or 0.5 mg protein/mL), rat intestine microsomes (final concentration of 0.5 mg protein/mL), or human liver microsomes (final concentration of 0.5 mg protein/mL) were mixed with 0.88 mM magnesium chloride, 4.4 mM saccharolactone, 0.022 mg/mL alamethicin, 15 μM test compound, and 3.5 mM UDPGA, which was added last. The mixture (final volume = 200 μL) was incubated at 37 °C for 1 h. An aliquot of 100 μL was taken, and the reaction was stopped by an addition of 50 μL acetonitrile containing 0.6% formic acid. The samples were vortexed for 30 s, centrifuged at 14,000× *g* rpm, and the supernatant was injected into UPLC system for analysis of OJT007 and metabolites. Genistein (15 μM) was used as a reference substrate for UGT. A negative control was prepared without the addition of cofactor.

### 3.7. Full Mass Scan and Product Scan Spectra for Matrix Free OJT007

First, a full scan (Q1 MS) in positive mode of the matrix free OJT007 was performed to identify OJT007’s protonated molecular ion [M+H]^+^. Subsequently, a product ion scan of OJT007 was conducted. The major product ion (P1) and the corresponding neutral losses were noted from the obtained results.

### 3.8. Identification of Phase I Metabolites

#### LC-MS/MS

After the biosynthesis of metabolites, the metabolite solution was analyzed by LC-MS/MS to identify oxidation metabolites. LC analysis was accomplished using a Shimadzu Nexera X2 UHPLC system (Columbia, MD, USA) coupled with a 4000 QTRAP^®^ triple quadrupole mass spectrometer system equipped with a Turbo Ion Spray ion source (AB Sciex. Redwood City, CA, USA) using the instrument conditions optimized in Section 3.3 The mass spectrometer strategy used Q1MI, product ion (MS2), and Neutral Loss (NL) to identify metabolites. 

Based on the data obtained from the product ion spectra of OJT007, a neutral loss scan was performed using the OJT007 microsome sample to screen metabolites. The first neutral loss (NL1) scan conducted corresponded to the major product ion of OJT007. The second neutral loss scan conducted was based on NL1+16 in order to screen for possible hydroxylated metabolites. As a final confirmation step, a product ion scan (MS2) was performed. The product ion scan of *m*/*z* 341, indicating mono-hydroxylated metabolites, was conducted. 

### 3.9. Identification of Phase II Metabolites

#### 3.9.1. LC-MS/MS

After biosynthesis of the glucuronide, the metabolite solution was analyzed by LC-MS/MS to identify glucuronide. The mass spectrometer used Q1MI and Neutral Loss (NL), and a product ion (MS2) scan was used to identify metabolites. The fragmentation of OJT007 was studied by using product ion scans in positive modes at a collision energy of 35 V. A constant neutral loss of 176 is characteristic of glucuronide metabolites; as such, a NL scan of 176 was conducted. In addition, a precursor ion scan for *m*/*z* 325 was conducted. 

#### 3.9.2. Hydrolysis by β-Glucuronidases

β-glucuronidases are enzymes that catalyze the hydrolysis of O- or S- glycosidic moieties, allowing for the liberation of aglycones from glycosides [32]. Following OJT007 biosynthesis of the glucuronide metabolite using liver microsomes for 2 h, the incubates were centrifuged at 14,000× *g* rpm for 15 min. The supernatant (200 μL) was added to 50 μL of the β-glucuronidase solution, which was prepared in KPI pH 7.4 at a concentration of 4000 units/mL. Then, the mixtures were incubated at 37 °C for 2 h to hydrolyze the glucuronide into parent compounds. The aliquots of the mixture were collected at 0, 1, and 2 h and then added into ice-cold 0.6% formic acid in acetonitrile solution to stop the reaction. The mixtures were then vortexed for 30 s and centrifuged at 14,000× *g* rpm for 15 min. The supernatant of this mixture was analyzed to obtain OJT007 and OJT007 glucuronide concentrations by a UPLC method. The control reaction consisted of an equivalent volume of KPI instead of the enzyme solution. The remaining glucuronide metabolite after hydrolysis was expressed as residual percent: the peak area of the analyte at different time points relative to the peak area of the same analyte at time zero multiplied by 100. The produced aglycone or unconjugated molecule was expressed as the concentration of the analyte at each time point. 

### 3.10. Determination of Molar Extinction Coefficient of OJT007 Glucuronide

Because of the lack of OJT007 glucuronide authentic standards, the OJT007 standard curve was used for the quantification of OJT007 glucuronide using a conversion factor (K). The determination of conversion factors has been described previously [27,30]. Briefly, an OJT007 standard curve was prepared in KPI without microsomes. The conversion factors were determined by comparing the UV absorption peak area of OJT007 and those of the glucuronide at an absorption wavelength of 343 nm by following its 100% conversion to a complete glucuronide. The difference in peak area of glucuronide metabolite (PA_c_) and peak area of OJT007 PA_o_ was calculated to be the ratio K. The average conversion factor (K) at three different concentrations (2, 10, and 50 µM) was used for estimating metabolite concentrations.

(1) $$K=\frac{{\mathrm{PA}}_{c}}{{\mathrm{PA}}_{o}}$$

The calculated conversion factor can be used to calculate the metabolite concentration using peak area of metabolite and the slope of the calibration curve obtained from parent compound (a1).

(2) $$C=\frac{{\mathrm{PA}}_{c}}{K\times a1}$$

### 3.11. Metabolic Stability Data Analysis

The half-life was calculated based on a single time point assay, as performed by others [23]. Briefly, the half-life was calculated based on first-order reaction kinetics using Equation (3), which allows the calculation of the percentage of the parent remaining at certain incubation times. For screening purposes, 60 min was selected as the optimal time for incubation as it would provide a higher predictive limit and would allow the differentiation of OJT007 microsomal stability.

(3) $$\mathrm{Half}-\mathrm{life}\;\left(t_{\frac{1}{2}}\right)=\frac{\ln2\times \left(\mathrm{Incubation}\;\mathrm{time}\right)}{\ln\left(\%\frac{\mathrm{Remaining}}{100}\right)}$$

### 3.12. Glucuronidation Metabolic Kinetics 

The kinetic parameters of OJT007 glucuronidation were determined by measuring the initial glucuronidation rates of OJT007 at 1 h after incubation with liver microsomes according to the glucuronidation assay described in Section 3.6. OJT007 concentrations evaluated ranged from 1.25 to 100 μM at a 0.035 mg of protein/mL. The glucuronidation rates were calculated as the amount of formed glucuronide per reaction time per protein amount (nmol/min/mg). Kinetic data were analyzed according to the Eadie–Hofstee plot for the selection of the appropriate equation [33]. If the Eadie–Hofstee plot was linear, the formation rates at different substrate concentration were fit to the standard Michaelis–Menten equation:

(4) $$V=\frac{V_{\max}\times C}{K_{m}+C}$$

where K_m_ is the Michaelis–Menten constant, and V_max_ is the maximum rate of glucuronidation. GraphPad Prism software (version 7.3 for Windows; GraphPad Software, La Jolla, CA, USA) was used. The visual inspection of fitted functions was used to select the best-fit enzyme kinetic model. 

## 4. Conclusions

Using rat liver microsomes, we identified three Phase I and one Phase II metabolites of OJT007. Using Q1 MI, neutral loss, and product ion scan in LC-MS/MS analysis, the three phase I metabolites were identified as mono-hydroxylation metabolites. In addition, by using LC-MS/MS analysis and chemical hydrolysis, the phase II metabolite was identified as an OJT007-mono-glucuronide. The Phase I microsomal stability assay indicated that OJT007 was relatively stable when incubated with rat liver microsomes. On the other hand, rapid metabolism via glucuronidation was observed, which may have implications in the in vivo disposition and bioavailability for OJT007. The early identification of OJT007 metabolic pathways will provide invaluable information during drug development process.

## Data Availability

The data presented in this study are available within this article.
